# Concordance of chlamydia infections of the rectum and urethra in same-sex male partnerships: a cross-sectional analysis

**DOI:** 10.1186/s12879-016-2141-7

**Published:** 2017-01-05

**Authors:** Vincent J. Cornelisse, Christopher J. Sherman, Jane S Hocking, Henrietta Williams, Lei Zhang, Marcus Y. Chen, Catriona S. Bradshaw, Clare Bellhouse, Christopher K Fairley, Eric P. F. Chow

**Affiliations:** 1 Melbourne Sexual Health Centre, Alfred Health, 580 Swanston St, Carlton, Melbourne, VIC 3052 Australia; 2 Central Clinical School, Faculty of Medicine, Nursing and Health Sciences, Monash University, Melbourne, Australia; 3 Melbourne School of Population and Global Health, University of Melbourne, Melbourne, Australia; 4 The Kirby Institute, The University of New South Wales, Sydney, Australia

**Keywords:** Chlamydia trachomatis [B03.440.190.190.190.750], Homosexuality [F01.145.802.975.500], Sexual Partners [M01.778], Disease Transmission, Infectious [N06.850.310], Chlamydia, Men who have sex with men, Transmission, Partnership, Couple, Dyad

## Abstract

**Background:**

Our study aimed to describe the concordance of chlamydia infections of the rectum and urethra in men who have sex with men (MSM) and their male partners.

**Methods:**

This was a cross-sectional study of chlamydia in MSM and their male sexual partners both attending Melbourne Sexual Health Centre (MSHC), Australia, between February 2011 and March 2015. We excluded partnerships where testing for chlamydia at both the rectum and urethra were not undertaken.

**Results:**

Our study included 473 partnerships (946 men). 30 men had urethral chlamydia, of whom 14 (47%, 95% CI 28 to 66) had a partner with rectal chlamydia. 46 men had rectal chlamydia, of whom 14 (30%, 95% CI 18 to 46) had a partner with urethral chlamydia. The proportion of men with rectal chlamydia when their partner had urethral chlamydia was significantly higher than the proportion of men with urethral chlamydia when their partner had rectal chlamydia (McNemar’s p = 0.02).

**Conclusions:**

This is the first study of chlamydia concordance in male sexual partnerships and suggests that transmission of chlamydia between the urethra and rectum may be less efficient than has been reported for transmission between the urethra and cervix in heterosexual couples. It also suggests that transmission from the urethra to the rectum may be more efficient than in the opposite direction.

**Electronic supplementary material:**

The online version of this article (doi:10.1186/s12879-016-2141-7) contains supplementary material, which is available to authorized users.

## Background

*Chlamydia trachomatis* remains one of the most common bacterial sexually transmissible infections (STI) worldwide [1, 2]. Chlamydia notifications have been increasing in Australia since the 1990s, and have doubled over the decade from 2004 to 2013, [3] due to both increased testing and a true increase in the prevalence of infection both in heterosexuals and men who have sex with men (MSM) [4–7]. New biomedical HIV prevention strategies such as pre-exposure prophylaxis and treatment as prevention will hopefully result in a significant reduction in new HIV transmissions, but there are concerns that the effective prevention of HIV will result in behavioural changes that will further increase the rates of bacterial STIs among MSM.

To address the rising rates of chlamydia, health departments have sought strategies to control this infection. These strategies include screening programmes, and such programmes have been evaluated in heterosexuals, but the most recent and largest clinical trial did not show a significant effect of screening on the prevalence of infection, even when annual screening rates reached 30% [8].

Screening programmes and other public health interventions can be evaluated using mathematical models. But to date no model of chlamydia transmission in MSM has been published, and there is a paucity of data on key information needed to develop such models. In MSM this information would include the natural duration of infection of the urethra and rectum, the transmission probability between these sites, and the potential role of chlamydia of the pharynx.

The aim of this study was to analyse the concordance of chlamydia infections at the rectum and urethra in male-male partnerships, and to use this data to infer the likelihood of transmission of chlamydia between the urethra and rectum within partnerships. Such information can be used to build mathematical models of chlamydia transmission, and may be useful for clinicians when counselling patients about their chlamydia infections.

## Methods

This was a cross-sectional study of chlamydia diagnoses of the urethra and rectum in MSM and their male sexual partners when both attended Melbourne Sexual Health Centre (MSHC) on the same day. MSHC is the major public sexual health clinic in Melbourne, Australia. It provides a free walk-in service and no referrals are required. Over the study period it provided approximately 35,000 consultations annually, and about 37% of consultations were for MSM [9]. All patients complete a computer-assisted self-interview (CASI) that collects their demographic details (e.g., age) and sexual behaviours (e.g., number of casual partners in the last three months, condom use with regular and casual partners during insertive and/or receptive anal sex in the last three months) [10]. From March 2011 onwards, CASI included the additional question “Is your partner also being seen today at this clinic?”. Patients who answered “yes” to this question were asked for their partner’s name. We initially included all MSM who answered yes to this question and whose partner we could identify, and who had testing for chlamydia between March 2011 and February 2015. Partners were paired and assigned a unique “partnership number”. MSHC uses an electronic medical record system in which clinicians enter a diagnosis code when a patient attends for a consultation.

We offered screening for chlamydia on rectal swabs and first void urine samples in all MSM, in line with Australian STI screening guidelines for MSM [11]. During the study period, the screening protocol at MSHC did not include testing for pharyngeal chlamydia. Chlamydia was tested using a strand displacement assay (Becton Dickinson, New Jersey, USA). Urethral and rectal chlamydia positivity was calculated as the number of positive tests divided by the number of tests performed.

All statistical analyses were performed using Stata (version 13.1, StataCorp LP). The 95% confidence interval (CI) for chlamydia positivity was calculated using the ‘exact’ binomial distribution [12]. The positivity of chlamydia results obtained at the urethra and rectum for each person in each partnership was paired and compared using the McNemar’s test of significance. Our primary analysis examined the concordance of chlamydia at the urethra and rectum, and was based on 473 partnerships in which both men were screened for urethral and rectal chlamydia on the same day. A secondary analysis was limited restricted to 406 partnerships where both men did not have symptoms of urethritis or proctitis, to remove possible selection bias as a result of the presence of symptoms as the primary reason of attending the clinic. Another secondary analysis was restricted to 352 partnerships who reported anal sex without condoms to exclude the protective effect of condom use.

## Results

During the study period, 1464 MSM attended MSHC with their partner on the same day, consisting of 732 partnerships, their demographics are described in Table 1. In 473 partnerships (946 men, 65% of total), both men were screened for urethral and rectal chlamydia on the same day. In 57 partnerships (114 men) at least one man had symptomatic urethritis, including 6 partnerships where both men had symptomatic urethritis. In 22 partnerships (44 men) at least one man had symptomatic proctitis, including two partnerships in which both men had symptomatic proctitis. There were 406 partnerships (812 men) in which both partners had complete testing for chlamydia and neither partner had symptomatic proctitis or urethritis (Additional file 1: Figure S1). Table 2 describes the frequency and proportion of chlamydia infections amongst these 946 men and 812 men respectively.

**Table 1:** Demographic and behavioural characteristics of the 1464 men attended the Melbourne Sexual Health Centre with their male partner on the same day

	Median (IQR)	Yes/total; % yes
Age (years)	28 (24 to 35)	
Age difference (years) between partners	4 (2 to 9)	
HIV positive		54/1464 (4%)
CSP in last 3 months (yes vs no)		702/963 (73%)
Condoms for RAI with RSP^a^		530/1330 (40%)
Condoms for RAI with CSP^a^		1078/1311 (82%)
Condoms for IAI with RSP^a^		508/1321 (38%)
Condoms for IAI with CSP^a^		1060/1309 (81%)
Number of CSP in last 3 months for those with CSP.	3 (1 to 5)	

*IQR* interquartile range, *RAI* receptive anal intercourse, *IAI* insertive anal intercourse, *RSP* regular sexual partner, *CSP* casual sexual partner

^a^Consistent condoms use at all times or no anal sex

**Table 2:** Chlamydia positivity in MSM, by anatomic site (individual MSM data, not grouped into partnerships), including only MSM from partnerships in which both men were tested for chlamydia at both rectum and urethra

	Positivity (all men) n/N; % (95%CI)	Positivity (excluding symptomatic cases) n/N; % (95%CI)
Chlamydia at any site	70/946; 7.4 (5.8 to 9.3)	48/812; 5.9 (4.4 to 7.8)
Urethral chlamydia	30/946; 3.2 (2.1 to 4.5)	18/812; 2.2 (1.3 to 3.5)
Rectal chlamydia	46/946; 4.9 (3.6 to 6.4)	35/812; 4.3 (3.0 to 5.9)
Both urethral and rectal chlamydia	6/946; 0.6 (0.2 to 1.4)	5/812; 0.6 (0.2 to 1.4)

N = men tested; *n* = positive tests; MSM = men who have sex with men

Of the 946 men, 30 had urethral chlamydia, of whom 14 (47%, 95% CI 28 to 66) had a partner with rectal chlamydia. Of the 946 men, 46 men had rectal chlamydia, of whom 14 (30%, 95% CI 18 to 46) had a partner with urethral chlamydia (Table 3). The proportion of men with rectal chlamydia when their partner had urethral chlamydia was significantly higher than the proportion of men with urethral chlamydia when their partner had rectal chlamydia (McNemar test; *p* = 0.02).

**Table 3:** Concordance of rectal chlamydia and urethral chlamydia between men in all 473 partnerships in which both men were screened for urethral and rectal chlamydia on the same day

	Urethra negative	Urethra positive	Total	Urethral chlamydia positivity % (95% CI)
Rectum negative	884	16	900	1.8 (1.0 to 2.9)
Rectum positive	32	14	46	30.4 (17.7 to 45.8)
Total	916	30	946	3.2 (2.1 to 4.5)
Rectal chlamydia positivity % (95% CI)	3.5 (2.4 to 4.9)	46.7 (28.3 to 65.7)	4.9 (3.6 to 6.4)	

(McNemar’s test *p* = 0.02)

Each man is represented twice: once as a urethra and once as a rectum

In a secondary analysis we excluded partnerships where either partner had symptomatic urethritis or proctitis. Of the 812 men in asymptomatic partnerships, 18 men had urethral chlamydia, of whom 8 (44%, 95% CI 22 to 69) had a partner with rectal chlamydia; and 35 men had rectal chlamydia, of whom 8 (23%, 95% CI 10 to 40) had a partner with urethral chlamydia (Table 4). The proportion of men with rectal chlamydia when their partner had urethral chlamydia was significantly higher than the proportion of men with urethral chlamydia when their partner had rectal chlamydia (McNemar test; *p* = 0.005). Table 5 includes data on 704 men who reported anal sex without condoms and proportions of chlamydia positivity are similar to those in Tables 3 and 4.

**Table 4:** Concordance of rectal chlamydia and urethral chlamydia between men in 406 partnerships in which both men did not have symptoms of urethritis or proctitis

	Urethra negative	Urethra positive	Total number of tests	Urethral chlamydia positivity % (95% CI)
Rectum negative	767	10	777	1.3 (0.6 to 2.4)
Rectum positive	27	8	35	22.8 (10.4 to 40.1)
Total number of tests	794	18	812	2.2 (1.3 to 3.5)
Rectal chlamydia positivity % (95% CI)	3.4 (2.3 to 4.9)	44.4 (21.5 to 69.2)	4.3 (3.0 to 5.9)	

(McNemar’s test *p* = 0.005)

Each man is represented twice: once as a urethra and once as a rectum

**Table 5:** Concordance of rectal chlamydia and urethral chlamydia between men in 352 partnerships who reported anal sex without condoms

	Urethra negative	Urethra positive	Total	Urethral chlamydia positivity % (95% CI)
Rectum negative	652	12	664	1.8 (0.9 to 3.1)
Rectum positive	27	13	40	32.5 (18.6 to 49.1)
Total	679	25	704	3.6 (2.3 to 5.2)
Rectal chlamydia positivity % (95% CI)	4.0 (2.6 to 5.7)	52.0 (31.3 to 72.2)	5.7 (4.1 to 7.7)	

(McNemar’s test *p* = 0.02)

Each man is represented twice: once as a urethra and once as a rectum

## Discussion

Our data provide the first estimate of the concordance of chlamydia infection between the rectum and the urethra in same-sex male partnerships. The data suggest that the likelihood of chlamydia transmission between men is lower than that reported between men and women. In a study of 494 heterosexual partnerships that included symptomatic individuals, chlamydia was found in 68% of men with an infected female partner, and in 70% of women with an infected male partner [13]. These male–female concordance rates are analogous to our 30.4% urethral chlamydia positivity in MSM with a partner with rectal chlamydia, and our 46.7% rectal chlamydia positivity in MSM with a partner with urethral chlamydia, respectively. The chlamydia concordance in these heterosexual couples is significantly higher than chlamydia concordance between MSM partners in our study, suggesting that the transmission probability between MSM is lower than between heterosexual men and women. This may explain why chlamydia is not much more common in MSM than in heterosexuals whilst other STIs such as gonorrhoea and syphilis are many times more common in MSM [14]. However, the comparison between heterosexuals and MSM is complicated by the fact that MSM are likely to have more sexual partners and concurrent sexual partners, as highlighted by the difference in sexual partner numbers in our study compared to that found in heterosexual couples [13].

There was only one partnership in which both men had urethral chlamydia in the absence of either man having rectal chlamydia (Additional file 1: Table S1a), which suggests that urethra-to-urethra transmission is uncommon. Similarly, there were only two partnerships in which both men had rectal chlamydia in the absence of either man having urethral chlamydia (Additional file 1: Table S1b), which suggests that rectum-to-rectum transmission is also uncommon. These findings also support the notion that pharyngeal-to-urethra and pharynx-to-rectum for chlamydia transmission is likely to be uncommon [15, 16].

We conducted secondary analyses to assess the effect of any possible bias that may have resulted from symptomatic presentations or condom use. As expected, when symptomatic presentations were excluded, the transmission probabilities were somewhat lower because including symptomatic presentations may have biased towards transmission having had occurred. When we included only men who practised condomless anal sex, the transmission probabilities were somewhat higher (33-52%) but still lower than had been reported for heterosexuals.

Our data showed that 47% of partners of men with urethral chlamydia had rectal chlamydia while only 30% of partners of men with rectal chlamydia had urethral chlamydia. This difference remained significant when the analysis was restricted by symptoms and condom use. While this cross-sectional study cannot determine the direction of chlamydia transmission within each partnership, the results suggest that transmission from the urethra to the rectum may be more likely than transmission from the rectum to the urethra. These data do not reflect a difference in transmission efficiency during a single exposure, but instead describe the cumulative result of all exposures. We did not further investigate the causes for this difference, but it may be that the mechanics of anal sex more efficiently facilitates urethra-to-rectum transmission than rectum-to-urethra transmission. The difference in concordance should not be due to a difference in bacterial loads, as chlamydia loads have been shown to be higher in the rectum than in the urethra [17]. Interestingly, the aforementioned analysis in heterosexual partnerships found equal bidirectional transmission of chlamydia between men and women, where 68% of the male partners of infected women had chlamydia, and 70% of female partners of infected men had chlamydia [13].

A limitation of our analysis is that chlamydia in the pharynx was not screened for in our study. It is plausible that men can acquire urethral or rectal chlamydia through penile-oral sex, oral-anal contact, or the use of saliva as a lubricant for penile-anal sex [18]; however, direct evidence for these specific modes of transmission is limited, and there is disagreement in the published literature on the role of pharyngeal chlamydia in overall transmission. Studies have generally shown that rates of pharyngeal chlamydia in MSM are low. A study of attendees at sexual health clinic in Amsterdam found that 1.1% of MSM (95% CI 0.9 to 1.3) tested positive for pharyngeal chlamydia, [15] which is low compared with a positive rate of 9.8% for rectal chlamydia and 4.4% for urethral chlamydia [15]. They also assessed rates of spontaneous clearance, and found that at a median follow-up time of 12 days (range 7–58) pharyngeal chlamydia had spontaneously cleared in 37.2% of MSM [15]. A 2014 study from Melbourne showed that urethral chlamydia infection in MSM was strongly associated with insertive penile-anal sex without condoms, whereas urethral gonorrhoea and primary syphilis was not, leading the authors to hypothesise that pharyngeal infections were significant contributors to transmission for urethral gonorrhoea and primary syphilis, but not for urethral chlamydia [19]. This observation is further supported by a study from Seattle that showed that only 3% of urethral chlamydia cases were attributable to oral sex, compared with 34% of urethral gonorrhoea cases [20]. In contrast, findings from a study of MSM attending a sexual health clinic in San Francisco in 2007 support the role of oral sex as a risk factor for urethral chlamydia. They found that men who reported penile-oral sex only, not penile-anal sex, in the three months before testing had a urethral chlamydia positive rate of 4.8% (95% CI 2.9-7.4), which was not significantly different from the rate of urethral chlamydia of 7.0% (95% CI 5.6 to 8.6) amongst men who reported insertive penile-anal sex without condoms in the three months before testing [21]. Similarly, the Sydney-based “Health In Men” (HIM) study found that occasional (but not frequent) insertive oral sex was a risk factor for urethral chlamydia in MSM [18]. Whilst we were not able to assess the role of pharyngeal chlamydia infection directly, our study found relatively few men who had chlamydia of both the urethra and rectum (<10%). One explanation for this observation is that the pharynx is perhaps not commonly involved in chlamydia transmission in MSM, as pharyngeal chlamydia could potentially transmit to both a partner’s urethra and rectum.

Our study has a number of other limitations. Firstly, we had no data on chlamydia infections in sexual partners other than the partner present on the day. Obviously, in every partnership at least one of the partners must have had sexual contact with a third party who had a chlamydia infection, but we are not able to determine in how many partnerships both partners separately acquired chlamydia from a third party, as this would require testing all third parties for chlamydia and conducting a molecular analysis of chlamydia serovars. A recently-published study of chlamydia concordance in heterosexual partnerships found that in 96.2% (51/53) of chlamydia-concordant partnerships both partners had the same chlamydia genotype (Schillinger et al., [22]). This finding suggests that concordance represents transmission within partnerships, but as described above, we may not be able to extrapolate from heterosexual partnerships to same sex male partnerships. By not accounting for instances where both partners acquired chlamydia from a third party, we may have over-estimated the likelihood of transmission between partners. Secondly, we did not collect data on the duration of the relationship, the frequency of sexual contact with that partner, whether their last sex act was with that partner, or whether they participated in group sex with that partner; hence it is not possible for us to estimate the per act transmission probability. Hence, in both our study and in the study by Quinn et al. of heterosexual partnerships, [13] chlamydia concordance reflects cumulative transmission rather than per-act transmission. Thirdly, we did not adjust for condom use because the number of men reporting consistent use of condoms for anal sex with their regular partner was quite small (171 of 946 men, 18%, for insertive anal sex; 172 of 946 men, 18%, for receptive anal sex). However, we did include a separate analysis of partnerships that reported anal sex without condoms with their regular partner, and found that the transmission estimates were similar (Table 5). Fourthly, our analysis included only men attending a single sexual health service with their male partner on the same day. It is likely that some men in partnerships did not present to our service on the same day as their partner and hence were not included in our study. We are not able to identify these partnerships, and their exclusion may have introduced a selection bias.

## Conclusions

These data provide an estimate of the relative probability of transmission of chlamydia between the urethra and rectum, which will assist in the development of mathematical models. These models may then be used to assess novel public health interventions to combat the rising rates of chlamydia infections amongst MSM. However, further work is needed to clarify the role of pharyngeal chlamydia infections in overall transmission rates. These data also suggest that transmission of chlamydia between the urethra and rectum is less common than has been reported for transmission between the urethra and cervix in heterosexual couples [13], which may explain why the rate of chlamydia is not significantly higher among MSM than among heterosexuals, unlike the rates of other bacterial STIs. However, this inference needs to be interpreted with caution, due to significant behavioural differences between MSM couples and heterosexual couples.

## Additional file

Additional file 1: Figure S1. Selection of cases. **Table S1a.** Urethral chlamydia in couples, after excluding couples with rectal chlamydia. Every MSM included as a “partner 1” and as a “partner 2”. **Table S1b.** Rectal chlamydia in couples, after excluding couples with urethral chlamydia. Every MSM included as a “partner 1” and as a “partner 2”. **Table S2.** Chlamydia positivity by site. Men who tested for rectal chlamydia, and their corresponding partner’s urethral chlamydia result. Symptomatic urethritis cases excluded. “Partner 2” includes only men did not have insertive penile-anal sex without condoms with casual and regular partners in the three months before testing. (DOCX 30 kb)

## Abbreviations

95% CI: 95% confidence interval
CASI: Computer-assisted self-interview
CSP: Casual sexual partner
IAI: Insertive anal intercourse
IQR: Interquartile range
MSHC: Melbourne sexual health centre
MSM: Men who have sex with men
RAI: Receptive anal intercourse
RSP: Regular sexual partner
STI: Sexually transmitted infection
