# Supplementary material for: Concordance of chlamydia infections of the rectum and urethra in same-sex male partnerships: a cross-sectional analysis
Source: BMC Infect Dis. 2017 Jan 5;17:22. doi: 10.1186/s12879-016-2141-7 (PMC5216557; doi:10.1186/s12879-016-2141-7)
Supplement: Additional file 1: Figure S1. — Selection of cases. Table S1a. Urethral chlamydia in couples, after excluding couples with rectal chlamydia. Every MSM included as a “partner 1” and as a “partner 2”. Table S1b. Rectal chlamydia in couples, after excluding couples with urethral chlamydia. Every MSM included as a “partner 1” and as a “partner 2”. Table S2. Chlamydia positivity by site. Men who tested for rectal chlamydia, and their corresponding partner’s urethral chlamydia result. Symptomatic urethritis cases excluded. “Partner 2” includes only men did not have insertive penile-anal sex without condoms with casual and regular partners in the three months before testing. (DOCX 30 kb) [file 12879_2016_2141_MOESM1_ESM.docx]

**Figure S1:** Selection of cases.

1464 MSM (732 partnerships) identified as attending with their partner for chlamydia testing

946 men (473 partnerships)

65% of 1464

832 men (416 partnerships)

57% of 1464

114 men (57 partnerships)

Excluded because one or both men had symptomatic urethritis.

518 men (259 partnerships)

Excluded because rectal or urethral testing not performed on one or both men.

**Table S1a:** Urethral chlamydia in couples, after excluding couples with rectal chlamydia. Every MSM included as a “partner 1” and as a “partner 2”.

|  | | Partner 2 | | | |
| --- | --- | --- | --- | --- | --- |
|  |  | Urethra negative | Urethra positive | Total | Percentage positive  % (95% CI) |
| Partner 1 | Urethra negative | 750 | 8 | 758 | 1.06%  (0.33 to 1.79) |
|  | Urethra positive | 8 | 2 | 10 | 20.0%  (-4.79 to 44.79) |
|  | Total | 758 | 10 | 768 | 1.30%  (0.50 to 2.10) |
|  | Percentage positive  % (95% CI) | 1.06%  (0.33 to 1.79) | 20%  (-4.79 to 44.79) | 1.30%  (0.50 to 2.10) |  |

**Table S1b:** Rectal chlamydia in couples, after excluding couples with urethral chlamydia. Every MSM included as a “partner 1” and as a “partner 2”.

|  | | Partner 2 | | | |
| --- | --- | --- | --- | --- | --- |
|  |  | Rectum negative | Rectum positive | Total | Percentage positive  % (95% CI) |
| Partner 1 | Rectum negative | 750 | 21 | 771 | 2.72%  (1.57 to 3.87) |
|  | Rectum positive | 21 | 4 | 25 | 0.16%  (1.41 to 1.73) |
|  | Total | 771 | 25 | 796 | 3.14%  (1.93 to 4.35) |
|  | Percentage positive  % (95% CI) | 2.72%  (1.57 to 3.87) | 0.16%  (1.41 to 1.73) | 3.14%  (1.93 to 4.35) |  |

**Table S2:** Chlamydia positivity by site. Men who tested for rectal chlamydia, and their corresponding partner’s urethral chlamydia result. Symptomatic urethritis cases excluded. “Partner 2” includes only men did not have insertive penile-anal sex without condoms with casual and regular partners in the three months before testing.

|  | | Partner 2 | | | |
| --- | --- | --- | --- | --- | --- |
|  |  | Urethra negative | Urethra positive | Total | Percentage positive  % (95% CI) |
| Partner 1 | Rectum negative | 282 | 4 | 286 | 1.40%  (0.04 to 2.76) |
|  | Rectum positive | 7 | 6 | 13 | 46.2%  (19.1 to 73.3) |
|  | Total | 289 | 10 | 299 | 3.34%  (1.3 to 5.38) |
|  | Percentage positive  % (95% CI) | 2.42%  (0.65 to 4.19) | 60.0%  (29.64 to 90.36) | 4.35%  (2.04 to 6.66) |  |
